# Supplementary material for: Chronic Inflammatory Status Observed in Patients with Type 2 Diabetes Induces Modulation of Cytochrome P450 Expression and Activity
Source: Int J Mol Sci. 2021 May 7;22(9):4967. doi: 10.3390/ijms22094967 (PMC8124164; doi:10.3390/ijms22094967)
Supplement: Supplementary file 1 [file ijms-22-04967-s001.zip › ijms-1189008-supplementary.pdf]

Supplementary Material:

Supplementary Table 1: Drugs mentioned in the paper with their clinical indications and P450 metabolism pathways.

Drugs	Clinical uses	Mechanism of action	P450 metabolism	References
Verapamil	1- Angina<br>2- Arrythmia<br>3- Hypertension	1- Relaxation and prevention of coronary artery spasm and reducing oxygen utilization<br>2- Decreasing the influx of calcium, prolonging the effective refractory period within the AV node and slows AV conduction<br>3- Decreasing systemic vascular resistance	CYP3A4, CYP2C9 and CYP1A2	[1]
Lidocaine	1- Production of local or regional anesthesia<br>2- Ventricular arrhythmias	1- stabilizes the neuronal membrane by inhibiting the ionic fluxes required for the initiation and conduction of impulses<br>2- Increasing the electric stimulation threshold of the ventricle during diastole	CYP1A2 and CYP3A4	[2,3]
Theophylline	1- Prophylaxis and chronic treatment of asthma<br>2- Acute exacerbations associated with asthma and other chronic lung diseases	Smooth muscle relaxation and suppression of the response of the airways to stimuli, by the inhibition of phosphodiesterase III	CYP1A2	[4,5]
Diclofenac	1- Osteoarthritis<br>2- Rheumatoid arthritis<br>3- Ankylosing spondylitis<br>4- Primary dysmenorrhea<br>5- Relief of mild to moderate pain	Prostaglandin synthetase inhibition	CYP2C9	[6]
Nateglinide	Improve glycemic control in adults with T2DM	Stimulating insulin secretion from the pancreas by interacting with ATP-sensitive potassium (K+ATP) channel on pancreatic beta-cells	CYP2C9	[7]
Phenytoin	1- Generalized tonic-clonic (grand mal) seizures<br>2- Complex partial seizures<br>3- Prevention and treatment of seizures occurring during or following neurosurgery	Causes voltage-dependent block of voltage gated sodium channels. This block sustains high frequency repetitive firing of action potentials.	CYP3A4, CYP2C9 and CYP2C19	[8]
Midazolam	1- Acute treatment of intermittent, stereotypic episodes of frequent seizure activity<br>2- Preoperative sedation/anesthesia/ anxiolysis/amnesia	Potentiation of GABAergic neurotransmission resulting from binding at the benzodiazepine site of the GABA receptor	CYP3A4	[9,10]

Glyburide	Improve glycemic control in adults with T2DM	Increasing the secretion of insulin from beta cells in the pancreas	CYP2C9	[11]
Repaglinide	Improve glycemic control in adults with T2DM	Stimulate the release of insulin from the pancreas by closing ATP-dependent potassium channels in the ß-cell membrane	CYP3A4 and CYP2C8	[12]
Bupropion	1- Treatment of major depressive disorder<br>2- Aid in smoking cessation treatment	Dual inhibition of norepinephrine and dopamine reuptake	CYP2D6, CYP2C19 and CYP2B6	[13]
Omeprazole	1- Treatment of duodenal ulcer and gastric ulcer<br>2- Treatment of GERD	Proton pump inhibitor, suppress gastric acid secretion by specific inhibition of the H+ /K+ ATPase enzyme system at the secretory surface of the gastric parietal cell	CYP3A4 and CYP2C19	[14]
Atorvastatin	1- Reduce elevated total-C, LDL-C<br>2- Reduce the risk of MI, stroke, revascularization procedures, and angina	Selective, competitive inhibitor of HMG-CoA reductase, the rate-limiting enzyme that converts 3-hydroxy-3 methylglutaryl-coenzyme A to mevalonate, a precursor of sterols, including cholesterol	CYP3A4	[15]
Nisoldipine	Treatment of hypertension	Dihydropyridine class of calcium channel antagonists, inhibits the transmembrane influx of calcium into vascular smooth muscle and cardiac muscle	CYP3A4	[16]
Cyclosporine	Prophylaxis of organ rejection in kidney, liver, and heart allogeneic transplants	Calcineurin-inhibitor immunosuppressant Inhibition of the production of cytokines involved in the regulation of T-cell activation. In particular, inhibits the transcription of interleukin 2.	CYP3A4	[17]
Tacrolimus	Prophylaxis of organ rejection in kidney, liver, and heart allogeneic transplants	Calcineurin-inhibitor immunosuppressant Binds to an intracellular protein, FKBP-12. A complex of tacrolimus-FKBP-12 is then formed, after which the phosphatase activity of calcineurin inhibited. As well as inhibiting the expression of several cytokines	CYP3A4	[18]
Clopidogrel	1- Acute coronary syndrome<br>2- Recent MI, recent stroke, or established peripheral arterial disease	P2Y12 platelet inhibitor, inhibitor of platelet activation and aggregation through the irreversible binding of its active metabolite to the P2Y12 class of ADP receptors on platelets	CYP2C19, CYP3A4, CYP2B6 and CYP1A2	[19]
Tolbutamide	Improve glycemic control in adults with T2DM	Increasing the secretion of insulin from beta cells in the pancreas	CYP2C9	[20]
Dextromethorphan	1- Temporarily relieve cough<br>2- PBA, in combination with quinidine sulfate	Sigma-1 receptor agonist and an uncompetitive NMDA receptor antagonist	CYP2D6	[21,22]

Chlorzoxazone	Skeletal muscle relaxant	Act at the spinal cord and subcortical levels of the brain to inhibit multisynaptic reflex arcs involved in producing and maintaining muscle spasms.	CYP2E1	[23]
Prasugrel	Reduction of thrombotic cardiovascular events in patients with acute coronary syndrome who are to be managed with PCI	P2Y12 platelet inhibitor, inhibitor of platelet activation and aggregation through the irreversible binding of its active metabolite to the P2Y12 class of ADP receptors on platelets.	CYP3A4, CYP2B6, CYP2C9 and CYP2C19	[24]
Ticagrelor	Reduction of thrombotic cardiovascular events in patients with acute coronary syndrome who are to be managed with PCI	P2Y12 platelet inhibitor, inhibitor of platelet activation and aggregation through the irreversible binding of its active metabolite to the P2Y12 class of ADP receptors on platelets.	CYP3A4	[25]

Abbreviations: ADP; adenosine diphosphate, AV; atrioventricular, C; cholesterol, GRED; gastroesophageal reflux disease, HMG-CoA; 3-*hydroxy-3-methylglutaryl-CoA*, MI; myocardial infarct, NMDA; N-methyl-D-aspartate, PBA; Pseudobulbar affect, PCI; percutaneous coronary intervention, T2D; type 2 diabetes

References

1. Available online: https://www.accessdata.fda.gov/drugsatfda_docs/label/2016/018925s010lbl.pdf Accessed on 01 April 2021.
2. Available online: https://www.accessdata.fda.gov/drugsatfda_docs/label/2010/006488s074lbl.pdf Accessed on 01 April 2021.
3. Bill, T.J.; Clayman, M.A.; Morgan, R.F.; Gampper, T.J. Lidocaine metabolism pathophysiology, drug interactions, and surgical implications. *Aesthet. Surg. J.* **2004**, *24*, 307–311.
4. Available online: https://www.accessdata.fda.gov/drugsatfda_docs/label/2011/022052s009lbl.pdf Accessed on 01 April 2021.
5. Barnes, P.J. Theophylline. *Pharmaceuticals (Basel)* **2010**, *3*, 725–747.
6. Available online: https://www.accessdata.fda.gov/drugsatfda_docs/label/2011/020142s021s022lbl.pdf Accessed on 01 April 2021.
7. Available online: https://www.accessdata.fda.gov/drugsatfda_docs/label/2017/021204s015lbl.pdf Accessed on 01 April 2021.
8. Available online: https://www.accessdata.fda.gov/drugsatfda_docs/label/2009/084349s060lbl.pdf Accessed on 01 April 2021.
9. Available online: https://www.accessdata.fda.gov/drugsatfda_docs/label/2017/208878Orig1s000lbl.pdf Accessed on 01 April 2021.
10. Lingamchetty, T.N.; Hosseini, S.A.; Saadabadi, A. Midazolam. StatPearls. In *Treasure Island (FL)*; 2021.
11. Available online: https://www.accessdata.fda.gov/drugsatfda_docs/label/2011/020051s017lbl.pdf Accessed on 01 April 2021.
12. Available online: https://www.accessdata.fda.gov/drugsatfda_docs/label/2012/020741s040lbl.pdf Accessed on 01 April 2021.
13. Foley, K.F.; DeSanty, K.P.; Kast, R.E. Bupropion: Pharmacology and therapeutic applications. *Expert Rev. Neurother*. **2006**, *6*, 1249–1265.
14. Shin, J.M.; Kim, N. Pharmacokinetics and pharmacodynamics of the proton pump inhibitors. *J. Neurogastroenterol. Motil*. **2013**, *19*, 25–35.
15. Lennernas, H. Clinical pharmacokinetics of atorvastatin. *Clin. Pharmacokinet*. **2003**, *42*, 1141–1160.
16. Available online: https://www.accessdata.fda.gov/drugsatfda_docs/label/2017/020356s027lbl.pdf Accessed on 01 April 2021.
17. Russell, G.; Graveley, R.; Seid, J.; al-Humidan, A.K.; Skjodt, H. Mechanisms of action of cyclosporine and effects on connective tissues. *Semin. Arthritis Rheum*. **1992**, *21* (Suppl. 3), 16–22.

18. Available online: https://www.accessdata.fda.gov/drugsatfda_docs/label/2018/210115s000,050708s047,050709s040lbl.pdf Accessed on 01 April 2021.).
19. Available online: https://www.accessdata.fda.gov/drugsatfda_docs/label/2010/020839s048lbl.pdf Accessed on 01 April 2021.
20. Babenko, A.P.; Gonzalez, G.; Bryan, J. The tolbutamide site of SUR1 and a mechanism for its functional coupling to K(ATP) channel closure. *FEBS Lett*. **1999**, *459*, 367–376.
21. Available online: https://www.accessdata.fda.gov/drugsatfda_docs/label/2010/021879s000lbl.pdf Accessed on 01 April 2021.
22. Schadel, M.; Wu, D.; Otton, S V.; Kalow, W.; Sellers, E.M. Pharmacokinetics of dextromethorphan and metabolites in humans: Influence of the CYP2D6 phenotype and quinidine inhibition. *J. Clin. Psychopharmacol*. **1995**, *15*, 263–269.
23. Ono, S.; Hatanaka, T.; Hotta, H.; Tsutsui, M.; Satoh, T.; Gonzalez, F.J. Chlorzoxazone is metabolized by human CYP1A2 as well as by human CYP2E1. *Pharmacogenetics* **1995**, *5*, 143–150.
24. Available online: https://www.accessdata.fda.gov/drugsatfda_docs/label/2019/022307s016lbl.pdf Accessed on 01 April 2021.
25. Dobesh, P.P.; Oestreich, J.H. Ticagrelor: Pharmacokinetics, pharmacodynamics, clinical efficacy, and safety. *Pharmacotherapy* **2014**, *34*, 1077–1090.
